# One year follow up for case series of myocarditis following mRNA vaccination against SARS-CoV-2

**DOI:** 10.1016/j.ahjo.2023.100294

**Published:** 2023-03-28

**Authors:** Matthew R. Petersen, Jeffery B. Budweg, Ralph M. Matar

**Affiliations:** a Department of Internal Medicine, University of Florida, 1600 SW Archer Rd, Room 4102, Gainesville, FL 32608, USA; b Division of Cardiovascular Medicine, University of Florida, Gainesville, FL 32608, USA

**Keywords:** Covid-19, Myopericarditis, Follow up, Myocarditis, mRNA vaccine

## Abstract

In 2021, the first rounds of modified mRNA vaccines against SARS-CoV-2 were tested and deployed. The vaccines themselves had great efficacy against severe infection, with rare and minimal side effects. One adverse effect that was reported, however, was incidence of myocarditis seen amongst mostly young males after their second vaccination dose. The disease course was self-limited. This study group published a case series in August of 2021 of four cases of this phenomenon. This paper is a followup to the original case series, providing an updated literature review and expert recommendations concerning the safety and benefits of the vaccines.

## Highlights

• Case series of 4 patients with SARS-CoV-2 vaccine-associated myocarditis, in whom, on follow up, had complete symptomatic and biomarker recovery.
• Multiple case series suggest a epidimiology feature of mostly young males affected by SARS-CoV-2 vaccine- associated myocarditis.
• Most cases were self resolving in course, without any known lasting physiological or structural cardiovascular effects.
• In review of totality of the evidence reviewed of this phenomina, evidence still supports vaccination in a risk/benefit analysis.

In 2021, to combat the deadly SARS-CoV-2 (COVID-19) pandemic, a novel form of vaccines was produced which utilize liposome-encapsulated recombinant mRNA that encodes the SARS-CoV-2 spike protein. In August 2021, this study group presented a case series of four patients believed to have COVID-19 vaccine-associated myocarditis. This follow up letter discusses the clinical follow up of these four patients, and highlights other relevant literature concerning SARS-CoV-2 vaccine associated myocarditis.

Four cases were presented involving a 20-year-old male, a 29-year-old male, a 30-year-old male, and a 23-year-old female, all of which presented with chest pain after their second dose of the COVID-19 mRNA vaccine. Table 1 details clinical findings concerning the four patients. All of the cases had elevated high sensitivity troponin levels and ECG changes. All had negative viral serologies, and all had resolution of symptoms within 3 days of admission and were discharged home [1]. Of the 4 patients described in our original paper, 3 completed short term follow up, while 1 was lost to follow up after hospitalization. All 3 patients who were able to follow up experienced complete resolution of their symptoms. 2 of the patients completed a course of 3 months of colchicine, after which they discontinued medications. All patients who followed up had normalization of inflammatory markers. Two patients had MRIs after discharge. Patient B's MRI, completed approximately 4 months after hospitalization, showed normal biventricular function with no areas of abnormal late gadolinium enhancement. Patient C's MRI, completed approximately 1 month after hospitalization, demonstrated a small area of subepicardial delayed myocardial enhancement suggestive of prior myocarditis with resolving edema.

**Table 1:** Summary of clinical finding at presentation and follow up.

	Age and sex	CRP (mg/L)	Peak troponin I HS (pg/mL)	Echocardiogram findings	ECG findings	Time to follow up (days)	Symptoms at follow up	CRP at follow Up (mg/L)	Follow up CMRI findings
A	23F	41	16,263	LVEF 55–60 %, basal inferior and basal inferolateral hypokinesis	Diffuse ST segment elevation with PR depressions	9	None	0.08	NA
B	20M	88	>27,000	LVEF 45 %, apical septal hypokinesis	Diffuse ST segment/J point elevations	13	None	2.5	Normal LV and RV size and systolic function, No abnormal LGE
C	29M	14	6802	LVEF 55 %, no regional wall motion abnormalities	Diffuse ST segment elevation with PR depressions	NA	None	NA	Normal LV and RV size and systolic function. Small area of subepicardial delayed enhancement of basal inferolateral wall. Suggestive of prior myocarditis with resolving edema
D	30M	129	2518	LVEF 65–70 %, no regional wall motion abnormalities	Inverted T waves in v4-v6	33	None	1.5	

CRP: C-reactive protein; ECG: electrocardiogram; TTE: transthoracic echocardiogram; LVEF: left ventricular ejection fraction; HS - high sensitivity; CMRI - cardiac magnetic resonance imaging; LV - left ventricle; RV - right ventricle.

Since vaccine rollout, there have been a significant number of reports to the Vaccine Adverse Event Reporting System (VAERS), with cases of pericarditis, myocarditis and myopericarditis in people who had received the vaccines. Large case series have also demonstrated myocarditis/pericarditis presenting most commonly in young males (under 21) after their second dose of a mRNA COVID vaccine. The vast majority of cases had a self-limiting course [2], [3].

Findings like these, as well as thousands of VAERS reports, encouraged a review by the Advisory Committee on Immunization Practices. This group concluded that there was an elevated risk of vaccination-associated myocarditis in males age 12–29, however, based on the low incidence and positive prognosis of these patients, the committee concluded that the benefits of vaccination far outweighed the risks [4].

In conclusion, our patients' experience was similar to those noted in other large case series [2], [3], with resolution of the inflammation and no known lasting effects. There have been several large case series, and many reports of this phenomenon to VAERS, again with similar findings of full recovery in the vast majority of cases. With the relatively low event rate and the overall good prognosis, with most patients experiencing a mild and self-limiting course, we believe that the benefits of the vaccines far outweigh the risks.

## Ethical approval

This manuscript has not been submitted for publication to any other online or in print journal. During the review process this manuscript will not be submitted for publication to any other entity. The writing, figures, and research was done originally by the authors, except where expressly noted in the manuscript and all appropriate references are cited.

Specifically for this manuscript submission, it is references a previously submitted manuscript to the Journal as cited below. We feel this is appropriate as it expands upon these authors previously published work. There was no outside industrial, professional or financial influence in the curation of this manuscript.

## Funding

There was no internal or external funding for this project.

## Declaration of competing interest

The authors declare that they have no known competing financial interests or personal relationships that could have appeared to influence the work reported in this paper.
